# Effect of wheat middling incorporation into wet corn distillers grains with solubles on apparent diet digestibility and ruminal fermentation characteristics in growing and finishing diets

**DOI:** 10.1093/tas/txae083

**Published:** 2024-05-14

**Authors:** Zachary M Duncan, Zachary L DeBord, Madison G Pflughoeft, William R Hollenbeck, Evan C Titgemeyer, K C Olson, Dale A Blasi

**Affiliations:** Department of Animal Sciences and Industry, Kansas State University, Manhattan, KS 66506, USA; Department of Animal Sciences and Industry, Kansas State University, Manhattan, KS 66506, USA; Department of Animal Sciences and Industry, Kansas State University, Manhattan, KS 66506, USA; Department of Animal Sciences and Industry, Kansas State University, Manhattan, KS 66506, USA; Department of Animal Sciences and Industry, Kansas State University, Manhattan, KS 66506, USA; Department of Animal Sciences and Industry, Kansas State University, Manhattan, KS 66506, USA; Department of Animal Sciences and Industry, Kansas State University, Manhattan, KS 66506, USA

**Keywords:** apparent digestibility, ruminal pH, starch, volatile fatty acids, wet distillers grains, wheat middlings

## Abstract

Two separate cross-over experiments were conducted to evaluate the effects of incorporating wheat middlings into wet corn distillers grains with solubles (WDGS) on apparent diet digestibility and ruminal fermentation characteristics in growing and finishing diets. In experiment 1, four ruminally cannulated heifers (313 ± 42.9 kg) were limit fed a high-energy growing diet that included WDGS (CON) or WDGS + wheat middlings (CON + WM) at 40% of diet dry matter (DM). The diet also contained (DM basis) 39.5% dry-rolled corn, 7.5% supplement, and 13% warm-season grass hay. In experiment 2, four ruminally cannulated Holstein steers (321 ± 17.4 kg) were fed a finishing diet that included WDGS (CON) or WDGS + wheat middlings (CON + WM) at 30% of diet DM. The diet also contained (DM basis) 60.3% dry-rolled corn, 2.7% supplement, and 7.0% warm-season grass hay. Experiments consisted of two 15-d periods that were conducted concurrently. Each period included 10 d of diet adaptation, 4 d of fecal collection, and 1 d of ruminal fluid collection. Fecal samples were collected on days 11 to 14 of each period and composite samples were analyzed for chromium to estimate apparent diet digestibility. On day 15, ruminal fluid samples were collected prior to feeding and again at 2, 4, 6, 8, 12, 18, and 24-h post-feeding. In experiment 1, DM, organic matter (OM), neutral detergent fiber (NDF), and acid detergent fiber (ADF) intake did not differ (*P* ≥ 0.11) between diets; however, starch intake was greater (*P* = 0.03) for heifers fed CON + WM compared with CON. Apparent DM, OM, NDF, and starch digestibilities were similar between diets (*P* ≥ 0.13), but feeding CON + WM tended to lead to lesser (*P* = 0.06) apparent ADF digestibility. Ruminal pH and total volatile fatty acid concentrations did not differ between diets (*P* ≥ 0.16); however, ruminal ammonia concentrations tended to be less (*P* = 0.09) for CON + WM compared with CON. In experiment 2, DM intake did not differ (*P* = 0.65) between diets. Apparent DM digestibility was greater (*P* = 0.01) for CON + WM compared with CON but the difference was small. Intake and apparent digestibility of OM, NDF, ADF, and starch did not differ (*P* ≥ 0.25) between diets. Ruminal ammonia concentrations were lesser (*P* = 0.03) while ruminal pH was greater (*P* = 0.02) for CON + WM compared with CON. Overall, incorporation of wheat middlings into WDGS had minimal impacts on feed intake, apparent diet digestibility, and ruminal fermentation characteristics when fed to growing and finishing cattle.

## Teaser

Feeding wet corn distillers grains with solubles and wheat middlings to growing and finishing cattle has been evaluated; however, the effects of incorporating wheat middlings into wet distillers grains to generate a novel co-product are unknown. Our data demonstrate feeding a co-product containing wet corn distillers grains with solubles plus wheat middlings had minimal effects on apparent diet digestibility and ruminal fermentation characteristics in a growing and finishing diet compared with feeding wet distillers grains alone.

## Introduction

Production of food and beverages for human consumption or ethanol from cereal grains generates byproducts that can be incorporated into livestock diets (Stock et al., 2000). A survey of consulting nutritionists indicated that grain byproducts were included in approximately 96% and 97% of receiving and finishing diets, respectively; moreover, wet corn distillers grains were the most commonly used grain byproduct in those types of cattle diets (Samuelson et al., 2016). Corn contains approximately 70% starch, and when starch is removed during ethanol production, the remaining protein, fat, fermentable fiber, and phosphorus are concentrated three-fold (Stock et al., 2000). Because wet corn distiller grains are relatively low in starch but high in digestible fiber, they can be used to provide dietary energy with a low risk of ruminal acidosis.

Wheat middlings may also be incorporated into beef cattle diets. Wheat middlings are a byproduct of flour milling and are defined as fine particles of wheat bran, germ, flour, and the offal from the “tail-of-the-mill” (AAFCO, 2022). Similar to wet corn distiller grains, wheat middlings contain elevated concentrations of crude protein, fermentable fiber, and minerals compared with wheat grain. Previous research demonstrated that wheat middlings can replace small portions of dry-rolled corn or alfalfa in finishing beef cattle diets without sacrificing growth performance (Dalke et al., 1997). Unfortunately, wheat middlings contain fine particles that have relatively low bulk density making them difficult to store and handle. They are often pelleted as a consequence, adding expense. One potential alternative to pelleting wheat middlings is to incorporate them into wet corn distillers grains with solubles (WDGS) to generate a new co-product (WDGS + WM). The objective of this experiment was to measure the effects of feeding WDGS or WDGS + WM on apparent diet digestibility and ruminal fermentation characteristics when included at 40% of diet dry matter (DM) in a growing diet and 30% of diet DM in a finishing diet. The growing diet used contained 39.5% dry-rolled corn and 13% roughage (DM basis) and was limit-fed once daily. The finishing diet used contained approximately 60% dry-rolled corn and 7% roughage (DM basis) and was fed for ad libitum intake.

## Materials and Methods

The Kansas State University Institutional Animal Care and Use Committee reviewed and approved all animal handling and animal care practices used in our experiments. All animal procedures were conducted in accordance with the Guide for the Care and Use of Animals in Agricultural Research and Teaching (FASS, 2020).

In experiment 1, four ruminally cannulated, crossbred Angus heifers (*n* = 4; initial body weight = 313 ± 42.9 kg) were limit-fed a high-energy growing diet that included WDGS (CON) or WDGS + WM (CON + WM) at 40% of diet DM (Table 1). Diets also contained 39.5% dry-rolled corn, 7.5% supplement, and 13% native-warm-season grass hay and were offered at 2.2% of body weight (DM basis) daily. In experiment 2, four ruminally cannulated Holstein steers (*n* = 4; initial body weight = 321 ± 17.4 kg) were fed a finishing diet that included either WDGS (CON) or WDGS + WM (CON + WM) at 30% of diet DM (Table 1). Diets also contained 60.33% dry-rolled corn, 2.67% supplement, and 7% native-warm-season grass hay and were fed for ad libitum intake. Diets were mixed using a Marion Paddle Mixer (model 2,030; Rapids Machinery Company; Marion, IA) and were fed once daily at 1000 hours. Animals were housed individually in soil-surfaced pens. Experiments were conducted simultaneously.

**Table 1.** Composition of experimental diets

	Experiment 1 (growing)		Experiment 2 (finishing)	
	CON	CON + WM	CON	CON + WM
Ingredient, % of DM				
Dry-rolled corn	39.50	39.50	60.33	60.33
Supplement 1^*^	7.50	7.50	—	—
Supplement 2^†^	—	—	2.67	2.67
WDGS^‡^	40.00	—	30.00	-
WDGS + WM^‡^	—	40.00	-	30.00
Warm-season grass hay	13.00	13.00	7.00	7.00
Composition, % of DM				
Dry matter, % as is	67.1	69.2	72.4	74.0
Crude protein	17.4	14.5	14.7	12.6
Organic matter	94.7	94.6	95.0	94.9
Ash	5.3	5.4	5.0	5.1
Neutral detergent fiber	23.1	22.3	16.8	16.2
Acid detergent fiber	9.2	9.2	6.1	6.0
Starch	30.7	34.0	44.0	46.5
Calcium	0.74	0.75	0.68	0.68
Phosphorus	0.48	0.59	0.45	0.54

^*^Supplement pellet formulated to contain (DM basis) 11.4% crude protein, 7% calcium, 0.65% phosphorus, 2.0% fat, and 338 mg/kg monensin (Rumensin; Elanco, Greenfield IN).

^†^Supplement formulated to contain (DM basis) 23.6% calcium, 2% phosphorus, 20.5% salt, 1,102 mg/kg monensin (Rumensin; Elanco, Greenfield IN).

^‡^WDGS: wet corn distillers grains with solubles; WDGS + WM: wet corn distillers grains with solubles + wheat middlings; Manufactured by PureField Ingredients; Russell, KS.

The following procedures were identical for both experiments. Treatments were arranged in a cross-over design utilizing two consecutive 15-d periods. Each period consisted of 10 d of diet adaptation, 4 d of fecal collection, and 1 d of ruminal fluid collection. On day 4 through day 14, 10 g of chromium oxide (Cr_2_O_3_) was administered intra-ruminally using a 24-mL gel capsule (Torpac; Fairfield, NJ). On day 11 through day 14, fecal grab samples were collected from each animal at 8-h intervals. Collection time advanced 2 h each day, so that each 2-h interval over 24 h was represented. Following collection, fecal samples were composited for each animal within period. Samples of individual feed ingredients were collected on day 10 through day 14 of each period. At the completion of the experiment, feed ingredient samples were composited within period.

On day 15, digesta samples were collected from four separate locations in the rumen prior to feeding and again 2, 4, 6, 8, 12, 18, and 24 h post-feeding. Following each collection, samples were strained through eight layers of cheesecloth, and ruminal pH was measured using a portable pH meter (Pinpoint; American Marine Inc., Ridgefield, CT). Strained ruminal fluid (1 mL) was subsequently pipetted into four 2-mL microcentrifuge tubes containing 250 µL of 25% (wt/vol) of *m*-phosphoric acid. After collection, ruminal fluid samples were immediately frozen at −20 °C pending further analysis.

Prior to the start of the experiment, a three-axial sensory accelerometer ear tag (Allflex Livestock Intelligence; Madison, WI) was applied in the right ear of each calf. Tags continuously measured rumination and activity, and data was summarized in 2-h increments. Activity was defined as all other movements excluding rumination. Data from days 10 to 14 of each period were used in the analysis. All data from one calf in experiment 2 were removed due to tag failure.

### Laboratory Analysis

At the completion of the experiment, feed ingredient and fecal samples were sent to a commercial laboratory (SDK Laboratories; Hutchinson, KS) for analysis. Samples were analyzed for DM, organic matter ([OM];100—ash; AOAC International, 1999), crude protein (*N* × 6.25; AOAC International, 1999), neutral detergent fiber (NDF; Van Soest et al., 1991), acid detergent fiber (ADF; Van Soest et al., 1991), starch (Richards et al., 1995), calcium (Bowers and Rains, 1988), and phosphorus (AOAC International, 1999) without replication.

To determine apparent diet digestibility, dried fecal material was ground using a 1-mm screen. Approximately 0.5 g of ground fecal material was subsequently placed in a muffle oven at 600 °C for 2 h. Chromium concentrations were then measured by atomic absorption spectrophotometry following preparation as described by Williams et al. (1962). Fecal chromium concentrations were used to estimate total fecal output and determine apparent diet digestibility according to Cochran and Galyean (1994). Ruminal fluid samples collected for volatile fatty acid (VFA) and ammonia analysis were centrifuged for 30 min at 17,000 × *g* at 4 °C. The supernatant was collected and VFA concentrations were measured using gas-liquid chromatography (Vanzant and Cochran, 1994). Ruminal ammonia concentrations in supernatant were measured as described by Broderick and Kang (1980).

### Statistical Analysis

All data were analyzed using the MIXED procedure in SAS (SAS 9.4, SAS Inst. Inc, Cary, NC). For intake and apparent digestibility, the model contained fixed effects of diet and period and a random effect of animal. Ruminal pH and concentrations of ruminal VFA and ruminal ammonia were analyzed as repeated measures. The model contained fixed effects of diet, period, hour, and diet × hour and a random effect of animal. The hour was the repeated term and the subject was animal × period. The covariance structure was spatial power for ruminal pH and autoregressive for ruminal ammonia concentrations and ruminal VFA concentrations as determined by Akaike information criterion statistics. No meaningful diet × hour interactions were observed for ruminal ammonia concentrations, ruminal VFA concentrations, or ruminal pH; therefore, the main effects of diet are presented.

Rumination and activity data were also analyzed as repeated measures. The model included fixed effects of diet, period, hour, and diet × hour and random effects of animal and day. Hour served as the repeated term and the subject was animal × period × day. The covariance structure was spatial power as determined by Akaike information criterion statistics. When protected by a significant *F*-test (*P* ≤ 0.05), treatment means were separated using the method of Least Significant Difference. Significance was declared at *P* ≤ 0.05 and tendencies at 0.05 ≤ *P* < 0.10.

## Results and Discussion

### Co-product Composition

The nutrient compositions of WDGS and WDGS + WM are presented in Table 2. Incorporation of wheat middlings into WDGS increased DM, starch, and phosphorus concentrations and reduced crude protein concentrations relative to WDGS alone. Cromwell et al. (2000) reported that wheat middlings contained an average of 89.6% DM, 16.2% crude protein, and 0.97% phosphorus (DM basis), whereas Buckner et al. (2011) indicated that WDGS contained an average of 32.5% DM, 31% crude protein, and 0.84% phosphorus (DM basis). In addition, wheat middlings were reported to contain approximately 19.5% more starch than WDGS (NASEM, 2016). Despite these differences, concentrations of OM, NDF, ADF, and calcium were similar between WDGS and WDGS + WM.

**Table 2.** Composition of WDGS and WDGS + WM

	Co-product	
	WDGS^*^	WDGS + WM^*^
Composition, % DM		
Dry matter, % as is	34.0	39.3
Crude protein	31.8	24.7
Organic matter	94.7	94.3
Ash	5.3	5.7
Neutral detergent fiber	24.2	22.2
Acid detergent fiber	7.9	7.8
Starch	3.2	11.6
Calcium	0.14	0.15
Phosphorus	0.82	1.11

^*^WDGS: wet corn distillers grains with solubles; WDGS + WM: wet corn distillers grains with solubles + wheat middlings; Manufactured by PureField Ingredients; Russell, KS.

### Experiment 1 Growing Diet

DM, OM, and ADF intake did not differ (*P* ≥ 0.64; Table 3) between CON and CON + WM. Intake of starch was greater (*P* = 0.03) for CON + WM compared with CON. Differences in starch intake reflected differences in the starch composition of the two feedstuffs. Apparent DM, OM, and starch digestibility did not differ (*P* ≥ 0.13; Table 3) between treatments in experiment 1; however, apparent ADF digestibility tended to be less (*P* = 0.06) for CON + WM compared with CON. Although NDF digestibility was not affected by treatment (*P* = 0.17), it should be noted that NDF digestibility followed a pattern similar to ADF digestibility. Liu et al. (2016) also observed a reduction in NDF and ADF digestibilities when wheat replaced corn and was fed at 66% of diet DM to crossbred beef cattle. Conversely, Zhu et al. (1997) reported apparent total-tract ADF digestibilities of wheat middlings were similar to wet corn gluten feed or a mix of dried distillers grains and hominy when diets were formulated to provide equal concentrations of NDF and were fed to lactating dairy cows. The reduction in apparent fiber digestibility in our experiment when heifers were fed CON + WM may have been associated with greater intake of starch. In addition, the NDF and ADF of wheat middlings may have been less digestible than NDF and ADF of WDGS.

**Table 3.** Effects of wet corn distillers grains with solubles or wet corn distillers grains with solubles + wheat middlings inclusion on intake, apparent digestibility, and ruminal fermentation characteristics in limit-fed growing heifers (experiment 1)

	Diet^*^			
Item	CON	CON + WM	SEM^†^	*P*-value^‡^
Number of observations	4	4		
Intake, kg/d				
Dry matter	6.98	7.00	0.100	0.87
Organic matter	6.61	6.62	0.094	0.94
Neutral detergent fiber	1.64	1.58	0.023	0.11
Acid detergent fiber	0.66	0.65	0.011	0.64
Starch	2.14	2.39	0.050	0.03
Apparent total-tract digestibility, %				
Dry matter	74.19	74.08	2.432	0.97
Organic matter	77.65	77.51	1.508	0.94
Neutral detergent fiber	60.22	54.27	2.794	0.17
Acid detergent fiber	61.53	51.35	2.580	0.06
Starch	93.37	96.43	1.201	0.13
Ruminal VFA^‖^, m*M*				
Acetate	39.91	36.56	2.196	0.13
Propionate	21.31	21.82	3.977	0.90
Butyrate	6.94	7.62	1.000	0.50
Valerate	1.50	1.92	0.644	0.51
Isobutyrate	0.62	0.65	0.066	0.59
Isovalerate	1.89	1.60	0.300	0.54
Acetate:propionate	2.08	2.00	0.384	0.86
Total VFA	72.16	70.17	8.744	0.70
Ruminal ammonia^‖^, m*M*	6.68	3.87	1.356	0.09
Ruminal pH^‖^	5.88	6.01	0.097	0.16
Rumination^$^, min/h	20.2	19.9	1.13	0.75
Activity^$^^,^^¶^, min/h	11.8	11.7	0.23	0.52

^*^CON: Wet corn distillers grains with solubles fed at 40% of diet dry matter (DM); CON + WM: Wet corn distillers grains with solubles + wheat middlings fed at 40% of diet DM.

^†^Mixed-model standard error of the mean (SEM) associated with comparison of treatment main-effect means.

^‡^Treatment main effect.

^‖^Average of values collected at 0, 2, 4, 6, 8, 12, 18, and 24 h post-feeding.

^$^No diet × hour interactions (*P* ≥ 0.70).

^¶^Movement excluding rumination.

Ruminal VFA concentrations for experiment 1 are presented in Table 3. Total VFA concentrations averaged across the 24-h sampling period did not differ (*P* = 0.70) between diets, and no effect of diet (*P* ≥ 0.13) was observed for ruminal concentrations of acetate, propionate, butyrate, valerate, isobutyrate, or isovalerate. Previous research demonstrated that limit-fed cattle consumed their daily feed allotment within 6 to 7 h of feed delivery (Schmidt et al., 2005; Duncan et al., 2022). In our experiment, ruminal VFA concentrations increased after feeding but began to decline approximately 6 h post-feeding (data not shown). Because heifers in experiment 1 were limit-fed at 2.2% of BW daily (DM basis), they consumed a large portion of their daily feed allotment within 6 h of feeding which is reflected by ruminal VFA concentrations decreasing after this time.

Ruminal ammonia concentrations tended to be greater (*P* = 0.09; Table 3) for CON compared with CON + WM. Differences in ruminal ammonia concentrations were associated with the composition of the co-products. Crude protein concentrations were 31.8% and 24.7% (DM basis; Table 2) for WDGS and WDGS + WM ingredients, respectively. As a result, dietary crude protein concentrations were 17.4% and 14.5% of diet DM (Table 1) for CON and CON + WM, respectively. Lower ruminal ammonia concentrations in CON + WM compared with CON could have slowed microbial growth and contributed to the reduction in apparent fiber digestibility. Although starch intakes were greater for heifers fed CON + WM, ruminal pH did not differ (*P* = 0.16; Table 3) between treatments. In addition, there was no effect of diet (*P* ≥ 0.52; Table 3) on time spent ruminating or heifer activity.

### Experiment 2 Finishing Diet

DM, OM, NDF, and ADF intakes did not differ (*P* ≥ 0.27; Table 4) between CON and CON + WM. Despite starch concentrations being 46.5% of diet DM in CON + WM and 44.0% of diet DM in CON, starch intake was similar (*P* = 0.66) between steers fed CON and steers fed CON + WM. VanderPol et al. (2006) reported that DM intake responded quadratically when WDGS replaced a blend of dry-rolled and high-moisture corn from 0% to 50% of diet DM and was maximized when WDGS were included at 30% of diet DM. To optimize feed intake, WDGS and WDGS + WM in our experiment were incorporated at 30% of diet DM.

**Table 4.** Effects of wet corn distillers grains with solubles or wet corn distillers grains with solubles + wheat middlings inclusion on intake, apparent digestibility, and ruminal fermentation characteristics in finishing steers (experiment 2)

	Diet^*^			
Item	CON	CON + WM	SEM^†^	*P*-value^‡^
Number of observations	4	4		
Intake, kg/d				
Dry matter	10.80	10.48	0.595	0.65
Organic matter	10.25	9.94	0.564	0.64
Neutral detergent fiber	1.85	1.72	0.092	0.27
Acid detergent fiber	0.67	0.64	0.038	0.48
Starch	4.74	4.88	0.282	0.66
Apparent total-tract digestibility, %				
Dry matter	78.06	78.44	0.027	0.01
Organic matter	79.42	79.83	0.253	0.25
Neutral detergent fiber	59.31	63.45	5.579	0.54
Acid detergent fiber	58.06	62.25	8.013	0.65
Starch	90.00	90.97	1.733	0.63
Ruminal VFA^‖^, m*M*				
Acetate	40.35	42.77	1.572	0.13
Propionate	31.77	24.98	3.676	0.07
Butyrate	6.57	9.44	0.577	< 0.01
Valerate	2.24	2.24	0.887	0.99
Isobutyrate	0.56	0.64	0.047	0.18
Isovalerate	0.95	1.88	0.731	0.21
Acetate:propionate	1.28	1.95	0.260	0.07
Total VFA	82.44	81.94	5.048	0.91
Ruminal ammonia^‖^, m*M*	4.62	2.61	0.879	0.03
Ruminal pH^‖^	5.53	5.71	0.071	0.02
Rumination^$^, min/h	21.5	21.0	0.87	0.57
Activity^$^^,^^¶^, min/h	16.0	16.5	0.38	0.16

^*^CON: Wet corn distillers grains with solubles fed at 30% of diet dry matter (DM); CON + WM: Wet corn distillers grains with solubles + wheat middlings fed at 30% of diet DM.

^†^Mixed-model standard error of the mean (SEM) associated with comparison of treatment main-effect means.

^‡^Treatment main effect.

^‖^Average of values collected at 0, 2, 4, 6, 8, 12, 18, and 24 h post-feeding.

^$^No diet × hour interactions (*P* ≥ 0.46).

^¶^Movement excluding rumination.

Apparent DM digestibility was greater (*P* = 0.01; Table 4) for CON + WM compared with CON; however, the difference in apparent DM digestibility was small (i.e., 0.38%) and not considered important. Apparent digestibilities of OM, NDF, ADF, and starch did not differ (*P* ≥ 0.25) between treatments. Apparent total-tract starch digestibility was 90.0% for CON and 91.0% for CON + WM, which was similar to results published by Owens and Zinn (2005) for feedlot steers consuming diets based on dry-rolled corn. In our diets, approximately 98% and 92% of starch intake for CON and CON + WM, respectively, was provided by dry-rolled corn.

Ruminal VFA concentrations for experiment 2 are presented in Table 4. Total VFA concentrations did not differ (*P* = 0.91) between treatments. There were no effects of diet (*P* ≥ 0.13) on average concentrations of ruminal acetate, valerate, isobutyrate, or isovalerate; however, ruminal propionate concentrations tended to be greater (*P *= 0.07) for CON compared with CON + WM. As a result, the acetate-to-propionate ratio tended to be less (*P* = 0.07) for CON compared with CON + WM. The trend for greater ruminal propionate concentrations with CON compared with CON + WM was unexpected. Ruminal propionate concentrations generally increase as dietary starch concentrations increase (Coe et al., 1999); however, in our experiment, dietary starch concentrations were greater for CON + WM compared with CON. In addition, ruminal butyrate concentrations in our experiment were greater (*P* < 0.01) for CON + WM compared with CON. Dalke et al. (1997) also observed a linear increase in ruminal butyrate concentrations when wheat middlings replaced portions of dry-rolled corn in a high-concentrate finishing diet based on dry-rolled corn.

Ruminal ammonia concentrations were greater (*P* = 0.03; Table 4) for CON compared with CON + WM. Similar to experiment 1, differences in ruminal ammonia concentrations were reflective of composition of the co-products. Crude protein concentrations were 12.6% of diet DM (Table 1) for CON + WM and 14.7% of diet DM for CON. In contrast to experiment 1, ruminal pH in experiment 2 was greater (*P* = 0.02; Table 4) for steers fed CON + WM compared with steers fed CON. There was no effect of diet (*P* ≥ 0.16; Table 4) on time spent ruminating or steer activity.

## Implications

Overall, incorporating wheat middlings with WDGS generated a co-product that contained more DM, starch, and phosphorus but less crude protein compared with WDGS alone. Because WDGS + WM contained more starch than WDGS, starch intake was greater for heifers limit-fed CON + WM compared with CON. Despite this circumstance, ruminal pH did not differ between diets. Although feeding WDGS + WM in a diet for ad libitum intake to finishing steers did not affect DM or starch intake relative to WDGS, ruminal pH was more acidic for steers fed WDGS. Overall, ruminal pH in both experiments was within the normal range for beef cattle fed grain-based diets and was interpreted to suggest that WDGS + WM can be fed at 40% of diet DM in growing diets or 30% of diet DM in finishing diets without inducing ruminal acidosis (Nagaraja and Titgemeyer, 2007).

Apparent DM, OM, NDF, and starch digestibilities were similar between growing and finishing calves fed diets containing WDGS and WDGS + WM. Apparent ADF digestibility tended to be lesser for growing heifers fed WDGS + WM compared with WDGS; however, there was no effect of diet on apparent fiber digestibility when WDGS + WM were fed to finishing steers. Feeding WDGS + WM to growing heifers did not influence ruminal VFA concentrations but did cause minor shifts in ruminal propionate and butyrate concentrations when fed to finishing steers. In addition, ruminal ammonia concentrations in both experiments were less in diets containing WDGS + WM compared with WDGS, reflecting the lower crude protein content of WDGS + WM compared to WDGS.

Wheat middlings are frequently pelleted because their physical characteristics make them difficult to store and handle. The pelleting process is expensive and can increase feed costs by $7.7 to $16.5 per metric ton (Dhuyvetter et al., 1999). Incorporating wheat middlings into WDGS would eliminate the cost associated with pelleting and could potentially improve wheat middling marketability. Based on these data, it appeared that feeding WDGS + WM to growing or finishing calves at 40% to 30% of diet DM, respectively, did not cause any major shifts in diet digestibility or ruminal fermentation characteristics and could be used as an alternative to pelleting.
